# MDA5 Can Be Exploited as Efficacious Genetic Adjuvant for DNA Vaccination against Lethal H5N1 Influenza Virus Infection in Chickens

**DOI:** 10.1371/journal.pone.0049952

**Published:** 2012-12-05

**Authors:** Matthias Liniger, Artur Summerfield, Nicolas Ruggli

**Affiliations:** Research Department, Institute of Virology and Immunoprophylaxis (IVI), Mittelhäusern, Switzerland; The Ohio State University, United States of America

## Abstract

Chickens lack the retinoic acid-inducible gene I (RIG-I) and sense avian influenza virus (AIV) infections by means of the melanoma differentiation-associated gene 5 product (chMDA5). Plasmid-driven expression of the N-terminal half of chMDA5 containing the caspase activation and recruitment domains [chMDA5(1-483)] triggers interferon-β responses in chicken cells. We hypothesized that mimicking virus infection by chMDA5(1-483) expression may enhance vaccine-induced adaptive immunity. In order to test this, the potential genetic adjuvant properties of chMDA5(1-483) were evaluated *in vivo* in combination with a suboptimal quantity of a plasmid DNA vaccine expressing haemagglutinin (HA) of H5N1 AIV. Co-administration of the HA plasmid with plasmid DNA for chMDA5(1-483) expression resulted in approximately 10-fold higher HA-specific antibody responses than injection of the HA plasmid mixed with empty vector DNA as control. Accordingly, compared with HA DNA vaccination alone, the chMDA5(1-483)-adjuvanted HA DNA vaccine mediated enhanced protection against a lethal H5N1 challenge infection in chickens, with reduced clinical signs and cloacal virus shedding. These data demonstrate that innate immune activation by expression of signaling domains of RIG-I-like receptors can be exploited to enhance vaccine efficacy.

## Introduction

Various vaccine strategies have been developed for the control of potential H5N1 highly pathogenic avian influenza A virus (HPAIV) pandemics [1], [2]. These approaches consist of inactivated viruses [3], live-attenuated viruses [4], [5], viral vector vaccines [6], [7], [8], [9] and DNA vaccines [10], [11], [12]. In general, live-attenuated viruses are the most effective viral vaccines (e.g. live oral poliovirus, measles virus, mumps virus, rubella virus and poxvirus). However, owing to safety concerns with live vaccine viruses, modern vaccine development focuses towards inactivated viruses, subunit vaccines, replication-deficient viral vector vaccines and DNA vaccines [13], [14]. With DNA vaccines in particular, the efficacy is rather low [15]. Therefore, significant efforts are invested in adjuvant development to increase vaccine efficacy and reduce production costs [16], [17]. One approach consists of co-expressing a protein with immunostimulatory properties, typically a cytokine [18], [19]. With the aim of developing a novel genetic adjuvant for avian influenza virus (AIV) vaccines in chickens, we sought to exploit the fact that innate immune activation triggers adaptive immune responses [20], [21], [22], [23]. The innate immune system uses pattern-recognition receptors (PRR) to sense the pathogen-associated molecular patterns (PAMP) that act as ‘danger signals’ associated with viral infections. PRR activation leads to an antiviral state mediated typically by type I interferon (type I IFN) induction and activation of antiviral proteins [24]. In this context, it was shown that PAMP recognition by Toll-like receptors (TLR) is involved in linking innate and adaptive immunity [25], [26], as selected TLR-agonists were found to enhance the efficacy of vaccines [27], [28], [29], [30]. The cytosolic RIG-I-like receptors (RLR), including the helicases retinoic acid-inducible gene I (RIG-I) and melanoma differentiation-associated gene 5 (MDA5), are the PRR that sense specifically intracellular viral RNA [24], [31], [32]. Activated RIG-I and MDA5 initiate a signaling involving the CARDIF (MAVS, VISA, IPS-1) adaptor, resulting in secretion of type I IFN [33], [34], [35], [36]. Importantly, the RLR signaling pathway was shown to contribute to the adjuvant activity of poly(I:C) [37]. A cell-permeable polypeptide containing sequences derived from the N-terminal caspase activation and recruitment domain (CARD) of CARDIF elicits type I IFN and possesses adjuvant properties [38]. Recently, CARDIF expression was found to enhance DNA-raised cellular immune responses and protection against H5N1 AIV infection in a mouse model [39]. Also, co-expression of RIG-I-specific PAMP augments humoral immune responses in DNA vaccinated mice [40]. These latter reports emphasize on the potential of RLR for vaccine and therapeutic applications (reviewed in reference [41]). When considering the RIG-I-like helicase pathway for the induction of innate immune responses in chicken, it must be noted that chickens lack RIG-I and sense dsRNA through chicken MDA5 (chMDA5) instead [42]. Accordingly, AIV infections in chicken cells are sensed by chMDA5, and type I IFN induction involves chicken LGP2, CARDIF and IRF3 [43]. Importantly, the same study also shows that in chicken DF-1 cells, plasmid-driven expression of the N-terminal half of chMDA5 results in the induction of the chIFN-β and the chMx promoters in the absence of PAMP. Expression of chCARDIF and chIRF3 does also activate the chIFN-β and chMx promoters, although to a lower extent. Considering this, we hypothesized that mimicking virus infection by the direct activation of the RIG-I-like signaling pathway through expression of the N-terminal domain of chMDA5 may enhance AIV vaccine-induced adaptive immune responses. We used a DNA vaccine against an H5N1 HPAIV as a model. A plasmid encoding the N-terminal 483 amino acids of chMDA5 [chMDA5(1-483)] co-administered with a plasmid expressing haemagglutinin (HA) of AIV significantly enhanced the HA-specific antibody responses, resulting in protection against H5N1 AIV challenge infection.

## Results

### Expression of chMDA5(1-483) Induces Type I IFN in Chicken Cells

**Figure 1:**
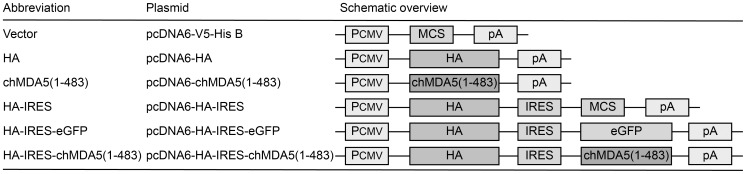
Schematic overview of the plasmid constructs used for DNA vaccination. The pcDNA6-V5-His B plasmid backbone (Vector) carrying a cytomegalovirus immediate early promoter (P_CMV_), a multiple cloning site (MCS) and the transcription termination and polyadenylation sequence (pA) was used for all protein expression constructs. The monocistronic expression plasmids pcDNA6-HA and pcDNA6-chMDA5(1-483) carry the HA gene of the H5N1 AIV Yamaguchi-7/04 and a cassette encoding the N-terminal 483 amino acids of the chicken MDA5, respectively. In the bicistronic expression plasmids pcDNA6-HA-IRES-eGFP and pcDNA6-HA-chMDA5(1-483), the IRES of the encephalomyocarditis virus was used to separate the HA gene from the eGFP and the chMDA5(1-483) gene, respectively. The plasmid pcDNA6-HA-IRES carries an empty MCS downstream of the IRES.

**Figure 2:**
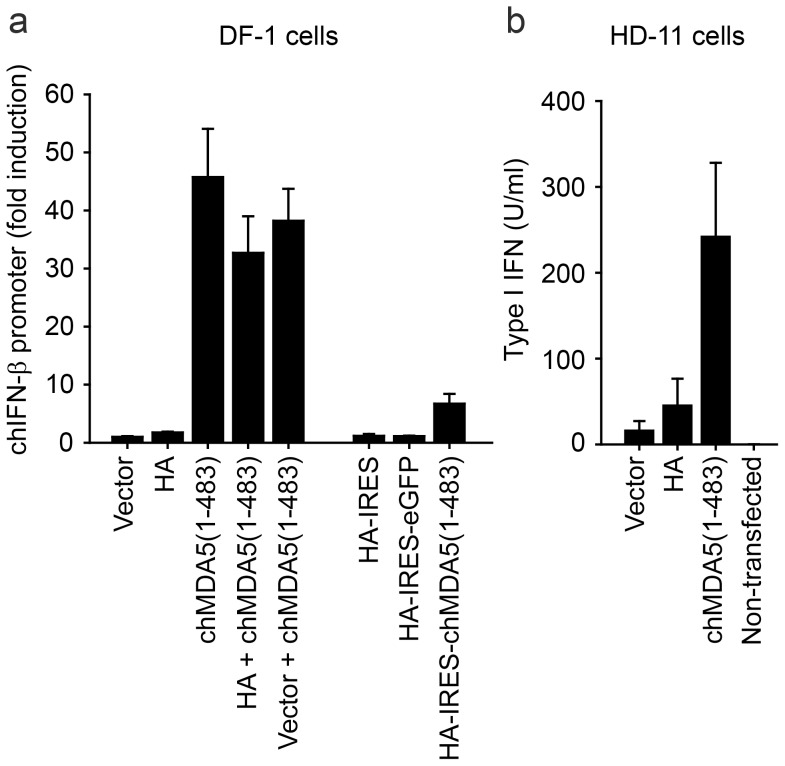
Expression of chMDA5(1-483) induces type I IFN. The effect of chMDA5(1-483) expression on the activation of the chIFN-β promoter (a) and on type I IFN secretion (b) was analyzed in the DF-1 fibroblast cell line (a) and in the HD-11 macrophage-like cell line (b), respectively. DF-1 cells were transfected with the reporter plasmids for measuring chIFN-β promoter activity and with the indicated expression constructs (a). The firefly and *Renilla* luciferase activities were determined 24 h later. The IFN-β promoter-induced firefly luciferase activity was normalized with the corresponding *Renilla* luciferase activity and expressed as fold induction compared to cells transfected with the empty expression plasmid (Vector). The bars represent the mean values of five independent transfections with the error bars showing the standard deviations. The graph is representative of two independent experiments. For type I IFN induction, HD-11 cells were transfected with the indicated plasmids (b). Eighteen hours later, the cell supernatants were assayed for type I IFN using the bioassay as described. The bars represent the mean values of six independent transfections with the error bars showing the standard deviations.

Overexpression of the N-terminal part of chMDA5 encompassing the CARD domains results in potent type I IFN induction [43]. Based on this, a truncated chMDA5 gene product [chMDA5(1-483)] was evaluated for its potential to augment vaccine-induced immune responses. According to the conserved domain database (CDD) at NCBI, the chMDA5(1-483) consists of two CARD repeats found typically in MDA5 proteins (CARD-MDA5-1, cd08818 and CARD-MDA5-2, cd08819; amino acids 7–94 and 108–195, respectively) and of a DEAD-like helicases superfamily domain (DEXDc, cd00046; from amino acid 316–483) including the ATP-binding region. An overview of the plasmid constructs and of the nomenclature used in the present study is provided in figure 1. Activation of the chIFN-β promoter mediated by the different plasmid constructs was analyzed in chicken DF-1 fibroblast cells (Fig. 2a). The expression of H5N1 influenza virus (A/chicken/Yamaguchi-7/04) HA alone did not induce the IFN-β promoter. As expected from previous studies [43], expression of chMDA5(1-483) resulted in IFN-β promoter-mediated luciferase expression in chicken DF-1 cells, which was not significantly impaired when the empty vector or the plasmid expressing HA were co-administered. The HA and chMDA5(1-483) proteins were also co-expressed from a bicistronic plasmid using an internal ribosomal entry site (IRES) of the encephalomyocarditis virus to separate the two genes (Fig. 1). When HA and chMDA5(1-483) were expressed from the same plasmid, a 10-fold lower chIFN-β promoter activity was obtained compared with expression from two separate plasmids (Fig. 2a). The chMDA5(1-483) induced a stronger chIFN-β promoter response than the twin CARD alone or the full-length chMDA5 (data not shown). Consistent with chIFN-β promoter activation, transfection of chicken macrophage-like HD-11 cells with the chMDA5(1-483) plasmid induced significantly higher levels of bioactive type I IFN than transfection with the vector or the HA plasmid alone (Fig. 2b). These results show that the expression of a polypeptide encompassing the N-terminal tandem CARD and the DEXDc domain of chMDA5 can be used to trigger type I IFN responses in the absence of PAMP.

### Co-administration of a Plasmid Expressing chMDA5(1-483) Augments HA DNA Vaccine-induced Antibody Responses in Chicken

**Figure 3:**
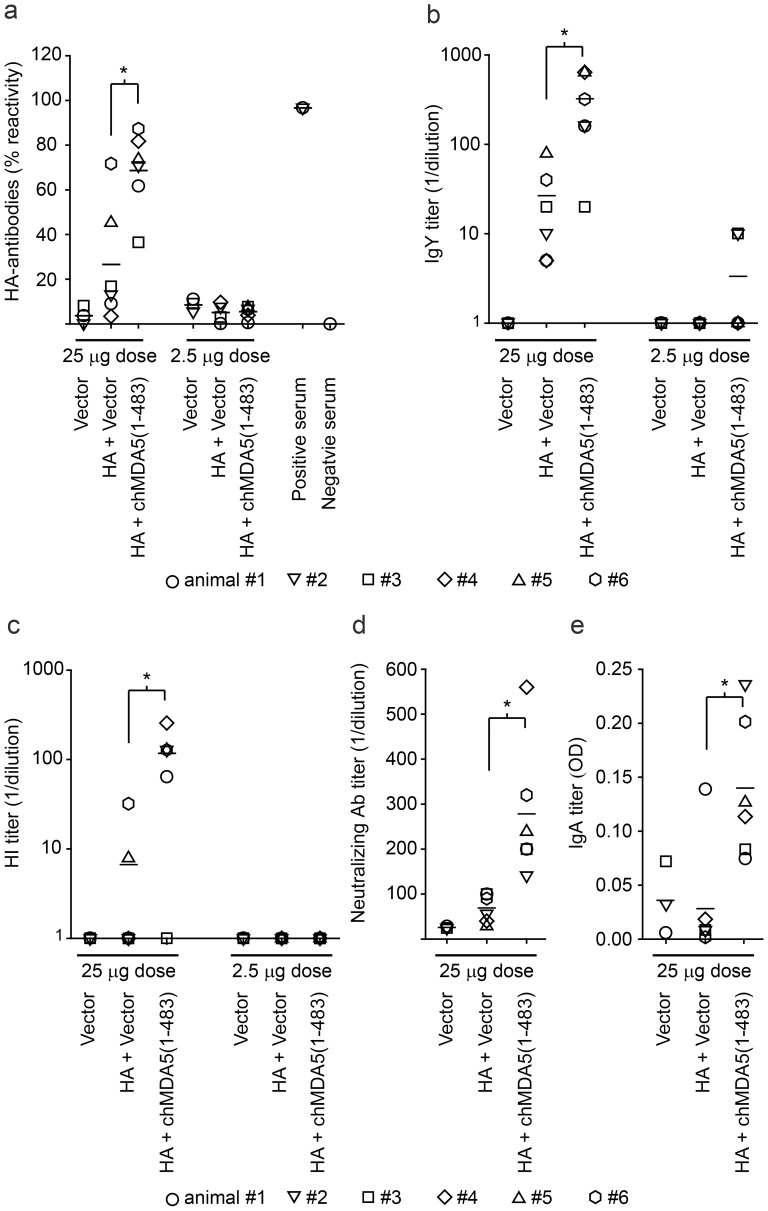
Effect of chMDA5(1-483) co-expression on HA-specific antibody responses elicited by HA DNA vaccination. Groups of 6 chickens were immunized with a plasmid DNA mixture containing equal amounts of the plasmids for HA expression and for chMDA5(1-483) expression. Doses of 25 µg or 2.5 µg of each plasmid DNA per animal were applied to two groups of 6 animals/dose, as indicated. The same two doses of plasmid DNA for HA expression alone mixed with an equal amount of empty expression plasmid were applied to two other groups of 6 chickens. Two control groups of 3 chickens each received 25 µg or 2.5 µg of empty expression plasmid each (Vector). Two immunizations were applied at an interval of 23 days. 20 days after the second vaccination (*i.e.* at 43 dpi), the chicken sera were tested for the presence of HA-specific antibodies. H5-specific antibodies were detected using a commercial competition ELISA. The reactivity of HA antibodies was calculated according the formula 100-[OD(Probe)/OD(Negative)x100] (a). Alternatively, the anti-HA IgY titers were determined in an indirect ELISA using immobilized recombinant HA protein and represented as reciprocal of the highest dilution that yielded an OD greater than 2.1 x of parallel preimmune serum samples (b). The HI titer was determined as the highest serum dilution resulting in complete inhibition of aggregation of chicken red blood cells caused by 8 HAU of Vac-1/04 (H5N1) virus (c). The neutralizing antibody titers in serum samples were determined with Vac-1/04 virus and plotted as reciprocal of the highest dilution resulting in 50% virus neutralization (d). The HA-specific IgA antibody content in the serum was measured by ELISA. Results are shown as absolute OD values from which the unspecific background values were subtracted (e). Each symbol represents an individual animal. The * indicates statistical significant differences calculated with the students t-test (p<0.05).

Considering the importance of innate immune activation for triggering adaptive immune responses, chMDA5(1-483) was analyzed for its adjuvant properties in the context of an experimental DNA vaccine against H5N1 HPAIV in chickens. To this end, two suboptimal doses of 25 µg or 2.5 µg of HA DNA vaccine per animal were mixed with equal amounts of plasmid DNA encoding chMDA5(1-483) or of the empty plasmid vector as control, and injected to groups of 6 chickens. Two groups of 3 animals were vaccinated with the two doses of empty plasmid vector as mock vaccination controls. 20 days after the booster vaccination (*i.e.* 43 days after the first immunization) the sera were analyzed for H5-specific antibody responses with different independent assays (Fig. 3). The chickens vaccinated with the high dose of HA DNA plasmid mixed with empty vector DNA responded with H5-specific antibodies as measured by competition (Fig. 3a) and indirect ELISA (Fig. 3b). This was also reflected in the haemagglutination inhibition (HI) assay (Fig. 3c) and in the virus neutralization assay using the heterologous Vac-1/04 H5N1 virus (Fig. 3d). However, no HA-specific serum IgA responses were induced (Fig. 3e), which is consistent with previous DNA vaccination studies [44]. Importantly, when chMDA5(1-483) DNA was co-administered with the HA DNA, significantly higher HA antibody responses were measured in all five tests, when compared with HA DNA alone. None of the animals vaccinated with the low dose of HA DNA in absence of chMDA5(1-483) showed any detectable immune responses in any of the tests performed. Nevertheless, a weak seroconversion was measured with the indirect ELISA in two animals that received chMDA5(1-483) with the low dose of HA DNA (Fig. 3b). Alternatively, groups of six chickens per group were immunized with 25 µg or 2.5 µg of bicistronic expression plasmids: the plasmid pcDNA6-HA-IRES-chMDA5(1-483) encoding HA and chMDA5(1-483) or the plasmid pcDNA6-HA-IRES-eGFP coding for HA and enhanced green fluorescent protein (eGFP) as control (Fig. 1). The bicistronic expression vectors did not induce any HA-specific antibodies in any of the immunized animals, irrespectively of the dosage (data not shown). It must be noted that three animals died for unknown reasons at day 26, 29 and 38 after immunization. Two of these animals were immunized with the low dose of HA DNA and one animal received the high dose of bicistronic HA-IRES-eGFP DNA. None of these latter chickens had received chMDA5(1-483)-adjuvanted vaccine.

### Co-administration of a Plasmid Expressing chMDA5(1-483) Enhances the Protective Efficacy of an HA DNA Vaccine Against HPAIV Infection in Chickens

**Figure 4:**
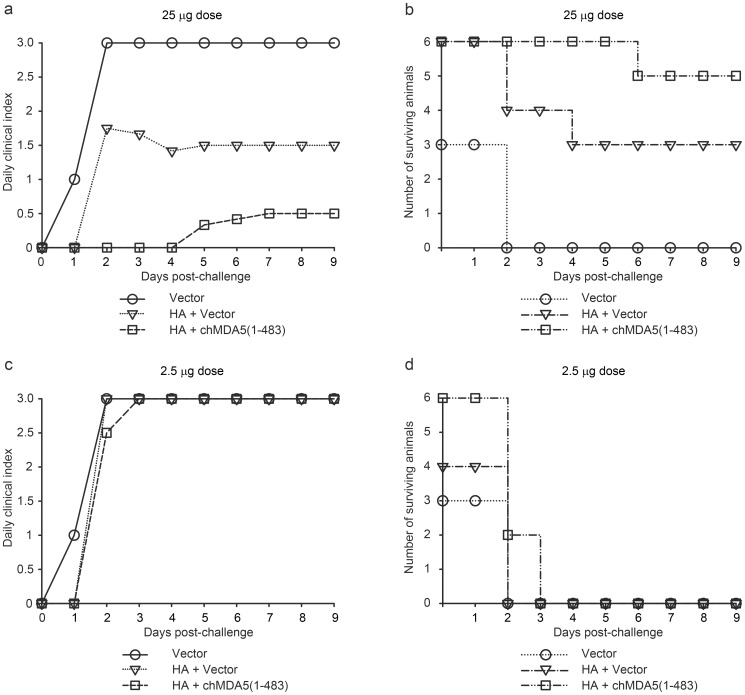
Effect of chMDA5(1-483) co-expressed with HA on clinical symptoms and survival after challenge with HPAIV H5N1. The chickens received two intramuscular immunizations with 25 µg (a and b) or 2.5 µg (c and d) of the indicated plasmid DNA at 23 days interval. After another 23 days (or at 46 dpi), the animals were challenged by intratracheal administration of the HPAIV Yamaguchi-7/04. The chickens were observed daily for clinical signs for a period of 9 days after the challenge infection. The daily clinical index was monitored as described elsewhere [58] with minor modifications: healthy (0), reduced activity (0.25), slightly ill (0.5), ill (1), severely ill (2), severely ill and euthanized (2.5) or dead (3). The daily clinical index is represented as the mean value of all chickens per group (a and c). The number of surviving animals is plotted against the time (in days) after the challenge (b and d). Note that the two groups (25 µg and 2.5 µg dose) vaccinated with control vector plasmid (Vector) consisted of only three animals each.

A reasonable humoral immune response against HA was obtained only when the HA antigen and the chMDA5(1-483) protein were expressed from two separate plasmids. Consequently, the protective capacity of the DNA vaccine was evaluated only with the chickens that were vaccinated with HA and chMDA5(1-483) expressed from the plasmid mixture at high and low dose. The challenge infection was performed with the HPAIV (H5N1) A/chicken/Yamaguchi-1/04 from which the HA gene was expressed by the DNA vaccine. In animals immunized with the empty vector, clinical signs of disease were observed one day after challenge, and all animals were dead or had to be euthanized in a moribund state on the second day after challenge infection (Fig. 4). Vaccination with 25 µg of plasmid DNA expressing HA mediated only limited protection, with first symptoms appearing two days after challenge (Fig. 4a, b). On this same day, one animal was dead and one severely ill animal had to be euthanized. A third chicken from this group had to be euthanized on day 4 after challenge. The other three animals recovered and survived the infection. With 25 µg of the chMDA5(1-483)-adjuvanted HA DNA vaccine, the chickens did not show any abnormal clinical symptoms, apart from one animal that became severely ill on day 5 and was euthanized on day 6 after challenge (Fig. 4a, b). None of the chickens were protected with the lower vaccine dose (Fig. 4c, d). Nevertheless, two animals that received 2.5 µg of chMDA5(1-483)-adjuvanted HA DNA vaccine survived for three days. These two animals were severely ill on day 3 after challenge and had to be euthanized. With 2.5 µg of the non-adjuvanted vaccine, none of the animals survived the second day after challenge. Note that in this latter group, only 4 animals were left at time of challenge (see above).

### The chMDA5(1-483)-adjuvanted HA DNA Vaccine Reduces Cloacal and Tracheal Virus Shedding after HPAIV Challenge Infection

An efficacious viral vaccine must reduce virus shedding to a minimum. In order to determine whether co-administration of the HA DNA vaccine and the plasmid expressing chMDA5(1-483) reduced shedding of challenge virus compared to vaccination with HA DNA alone, tracheal and cloacal swabs were collected daily for a period of nine days following infection with H5N1 AIV from the animals vaccinated with 25 µg of DNA. On day 2 after challenge, all control and vaccinated animals that died or were euthanized had comparable amounts of tracheal and cloacal viral RNA (Tables 1 and 2). The animals that survived the challenge infection had lower quantities of viral RNA in the trachea (Table 1). However, the tracheal swabs were virus positive for all except one animal on the first and second day after challenge, which may in part be due to the intratracheal infection. The chickens immunized with the chMDA5(1-483)-adjuvanted HA vaccine were negative for viral RNA one day earlier than the chickens that received HA DNA only. Importantly, no or only traces of viral RNA were detected in cloacal samples collected from the chickens that received the chMDA5(1-483)-adjuvanted HA DNA vaccine (Table 2). There was a marked difference when compared with the group of animals that were vaccinated with HA DNA alone. Interestingly, the only animal (chicken #1) that had to be euthanized on day 6 after challenge despite vaccination with chMDA5(1-483)-adjuvanted HA DNA started to shed viral RNA in the cloaca on day 4 after challenge only, when the first symptoms appeared. The reason for the delayed infection and lack of protection of this single animal of the group is unknown. The lack of protection was not related to a lower antibody response. In conclusion, these data show that co-expression of chMDA5(1-483) enhances the efficacy of a HA DNA vaccine and reduces virus shedding substantially.

**Table 1:** Quantification of viral RNA in tracheal swabs from animals vaccinated with 25 µg of DNA.

Plasmid(s)	Animal	1 dpc	2 dpc	3 dpc	4 dpc	5 dpc	6 dpc	7 dpc	8 dpc	9 dpc
Vector	#1	++	+++++†	†	†	†	†	†	†	†
	#2	++	+++++†	†	†	†	†	†	†	†
	#3	+++	+++++†	†	†	†	†	†	†	†
HA+Vector	#1	+++	++++†	†	†	†	†	†	†	†
	#2	++	+++	(+)	++	–	–	–	–	–
	#3	+	+	(+)	(+)††	†	†	†	†	†
	#4	+	+++++††	†	†	†	†	†	†	†
	#5	(+)	++	++	(+)	–	–	–	–	–
	#6	-	+++	+	+	–	–	–	–	–
HA+chMDA5(1-483)	#1	++	++	+	++	–	–††	†	†	†
	#2	(+)	++	–	–	–	–	–	–	–
	#3	(+)	+++	(+)	–	–	–	–	–	–
	#4	–	–	–	–	–	–	–	–	–
	#5	+	++	–	–	–	–	–	–	–
	#6	+	+++	++	–	–	–	–	–	–

Abbreviations: dpc, days post-challenge; -, PCR negative; RNA copies per 100 µl swab, (+) <10^3^,+<10^4^,++<10^5^,+++<10^6^,++++<10^7^,+++++<10^8^;

† animal dead;

†† animal euthanized.

**Table 2:** Quantification of viral RNA in cloacal swabs from animals vaccinated with 25 µg of DNA.

Plasmid(s)	Animal	1 dpc	2 dpc	3 dpc	4 dpc	5 dpc	6 dpc	7 dpc	8 dpc	9 dpc
Vector	#1	+++	++++†	†	†	†	†	†	†	†
	#2	+++	++++†	†	†	†	†	†	†	†
	#3	+++	+++++†	†	†	†	†	†	†	†
HA+Vector	#1	+++	+++†	†	†	†	†	†	†	†
	#2	–	+	–	–	–	–	–	–	–
	#3	–	–	–	–††	†	†	†	†	†
	#4	++	++++††	†	†	†	†	†	†	†
	#5	–	+++	–	–	–	–	–	–	–
	#6	–	–	–	–	–	(+)	–	–	–
HA+chMDA5(1-483)	#1	–	–	–	++++	(+)	–††	†	†	†
	#2	–	–	–	–	–	–	–	–	–
	#3	–	(+)	(+)	–	–	–	–	–	–
	#4	–	–	–	–	–	–	–	–	–
	#5	–	–	–	–	–	–	–	–	–
	#6	–	–	–	–	–	–	–	–	–

Abbreviations: dpc, days post-challenge; -, PCR negative; RNA copies per 100 µl swab, (+) <10^3^,+<10^4^,++<10^5^,+++<10^6^,++++<10^7^,+++++<10^8^;

† animal dead;

†† animal euthanized.

## Discussion

One major challenge in the development of new generation vaccines is to overcome the general limited efficacy compared to classical live attenuated vaccines. DNA and RNA vaccines in particular require several booster injections and potent adjuvants to be protective. The increasing knowledge of the functions of the innate and adaptive immune system led to the discovery of novel immunomodulating agents such as cytokines or TLR agonists that may be exploited to improve the adaptive immunity induced by a vaccine [23], [30], [45], [46]. In a previous study, the function of the chicken RIG-I-like helicase MDA5 in the perception of AIV infections was demonstrated [43]. The present study shows that the expression of the N-terminal 483 amino acids of the chicken MDA5 [chMDA5(1-483)] enhances the humoral immune responses and the protective immunity of a DNA vaccine expressing HA of HPAIV. The overexpression of chMDA5(1-483) induces type I IFN in the absence of viral triggers. Consequently, the potential of chMDA5(1-483) as a genetic adjuvant was evaluated in the context of a DNA vaccine expressing the HA glycoprotein of AIV H5N1 applied at a suboptimal dose in chickens. Co-administration of a plasmid expressing chMDA5(1-483) and a plasmid expressing HA resulted in significantly higher antibody responses and in enhanced protection against a homologous lethal H5N1 challenge infection, with reduced virus shedding when compared with the HA plasmid alone. The reasons for the lack of protection in one out of six animals that had received the adjuvanted HA DNA vaccine is unknown. The delayed appearance of symptoms and virus shedding may be the result of natural infection with a particularly high viral load due to prolonged close contact with severely ill animals and with heavily contaminated feces. Challenge against a heterologous H5N1 strain was not assessed. However, the virus neutralization data with the LPAIV Vac-1/04 strain (Fig. 3d) suggest that chMDA5(1-483) expression may also enhance the protective effect against heterologous infections.

The effect of chMDA5(1-483) expression on cellular immune responses was not assessed. In a related study, the RLR adaptor molecule VISA (CARDIF) enhanced the cellular immune responses but not the humoral immunity in mice [39]. The lack of efficient stimulation of humoral immune responses by VISA in the latter study may be due to the limited capacity of VISA to induce innate immune responses, compared with the N-terminal domain of RLR [43]. Recently also, the cytosolic DNA sensor DNA-dependent activator of interferon regulatory factors (DAI) was shown to function as a genetic adjuvant for enhanced adaptive T cell immunity in mice [47]. The RLR pathways and the induction of antiviral innate immune responses are generally conserved between birds and mammals [48], [49], with the exception of the RIG-I deficiency in chickens [50]. Similar to observations in chickens [43], expression of twin CARD-containing polypeptides derived from mammalian RLR results in the induction of type I IFN in mammalian cells [51]. Therefore, it can be speculated that the mammalian counterpart of the N-terminal half of MDA5 used in the present study may also exert adjuvant functions in mammalian hosts. These domains of MDA5 may also enhance the efficacy of mRNA vaccines, of viral replicon vector vaccines and of alternative DNA, RNA or protein delivery systems including nanoparticles, liposomes or virosomes, which needs to be explored in future studies.

## Materials and Methods

### Ethics Statement

The animal experiments were performed in compliance with the Swiss animal protection law and approved by the animal welfare committee of the canton of Berne, Switzerland (authorization number 101/06).

### Cells

The immortalized chicken embryonic fibroblast cell line DF-1 (ATCC, No. CRL-12203) was propagated in DMEM+GlutaMAX-I (Life Technologies) supplemented with 10% heat-inactivated fetal bovine serum (FBS, BioWest). The chicken macrophage-like HD-11 cells were kindly provided by Peter Stäheli (Department of Virology, University of Freiburg, Germany) and cultivated in DMEM+GlutaMAX-I supplemented with 2% chicken serum (Invitrogen) and 8% heat-inactivated FBS. Madin-Darby canine kidney (MDCK) cells were propagated in MEM supplemented with 10% FBS, nonessential amino acids, and 1 mM sodium pyruvate (Life Technologies).

### Viruses

The highly pathogenic avian H5N1 virus A/chicken/Yamaguchi/7/04 (Yamaguchi-7/04, GenBank AB166859 to AB166866) [52] was employed for the challenge study in chicken. HI assays were performed with the low pathogenic H5N1 vaccine strain A/Vac-1/Hokkaido/04 (Vac-1/04, GenBank AB259709 to AB259716) [53]. The viruses were generated by reverse genetics and the stocks were prepared from infected embryonated chicken eggs [54]. Titers were determined by end-point titrations on MDCK cells [55].

### Construction of Plasmids and Preparation of DNA Vaccines

All preparative PCR reactions were performed with Accuprime Pfx DNA polymerase (Life Technologies). The BamHI and SmaI fragment containing the IRES from the bicistronic vector pIRES1-neo (Takara Bio Europe/Clontech) was cloned into the BamHI and EcoRV sites of the eukaryotic expression vector pcDNA6-V5-His B (Life Technologies), resulting in pcDNA6-IRES. In order to introduce the HA gene of the H5N1 Yamaguchi-7/04 virus into pcDNA6-IRES, PCR was performed with the oligonucleotides Y-HA-F (TATAGCTAGCCACCATGGAGAAAATAGTGCTTCTTCTTG) and Y-HA-R (TCATGGATCCTTAAATGCAAATTCTGCATTGTAACG) using the pHW2000-derived plasmid carrying the Yamaguchi-7/04 HA segment (GenBank AB166862.1) as template. The PCR fragment was ligated into the NheI and BamHI sites of pcDNA6-IRES to obtain the plasmid pcDNA6-HA-IRES. For the construction of the plasmid pcDNA6-HA-IRES-GFP a fragment encoding eGFP from pEGFP-C2 (Takara Bio Europe/Clontech) was amplified by PCR using the oligonucleotides GFP-F (TATACTCGAGATGGTGAGCAAGGGCGAGGAG) and GFP-R (TCATACCGGTTCACTTGTACAGCTCGTCCATGCC). The GFP PCR product was subcloned into pcDNA6-HA-IRES using the restriction sites XhoI and AgeI. For the construction of plasmid pcDNA6-HA-IRES-chMDA5(1-483), a gene cassette encoding the first 483 amino acids of chMDA5 was amplified using the oligonucleotides chMD-F (TATACTCGAGATGTCGGAGGAGTGCCGAGAC) and N-DEXDc-R (TCATACCGGTTCAAGGTGAGGCTGTAAGTCCCAG) and the plasmid pcDNA6-chMDA5 [43] as a template, and inserted into the plasmid pcDNA6-HA-IRES using the restriction endonuclease sites XhoI and AgeI. The monocistronic expression plasmid pcDNA6-chMDA5(1-483) was generated by transfer of the chMDA5(1-483) cassette from pcDNA6-HA-IRES-chMDA5(1-483) into pcDNA6-V5-His B using the NheI and BamHI restriction sites. Plasmid pcDNA6-HA was constructed by subcloning the XhoI and AgeI fragment released from pcDNA6-HA-IRES-chMDA5(1-483) into the corresponding sites of pcDNA6-V5-His B. The plasmids were propagated in *E.coli* XL1-Blue (Agilent Technologies), and endotoxin-free plasmid DNA was prepared by anion-exchange chromatography using the NucleoBond Xtra Midi EF DNA extraction and purification system (Macherey-Nagel). The nucleotide sequences of the plasmid inserts were verified by automated DNA sequencing using the ABI 3130 Genetic Analyzer (Life Technologies).

### Chicken IFN-β Promoter Reporter Assay and Chicken Type I IFN Bioassay

The chicken IFN-β promoter activities were determined using a transient IFN-β promoter reporter assay described earlier [43]. Briefly, chicken DF-1 cells were seeded in 96-well plates (2–4×10^4^ cells/well) and co-transfected with the monocistronic or bicistronic pcDNA6-V5-His B-derived expression vectors (50 ng/well) and with 50 ng/well pGL3-P-chIFN-β-luc and 0.1 ng/well phRL-SV40 (Promega) reporter plasmids using Fugene HD (Roche Applied Science). The cells were lysed with 20 µl of 1X passive lysis buffer (Promega) 24 h after transfection. The samples were assayed for firefly and *Renilla* luciferase activities using the Dual-Luciferase Reporter Assay System (Promega) and a Centro LB 960 luminometer (Berthold Technologies). The type I IFN bioactivity in the supernatants of transfected HD-11 cells was measured with a Mx promoter-driven luciferase reporter bioassay in CEC-32 cells, as described elsewhere [56], [57]. Briefly, chicken HD-11 cells were seeded in 96-well plates and transfected with pcDNA6-V5-His B-derived expression vectors (50 ng/well) using Fugene HD. Eighteen hours after transfection, the cell supernatants were assayed for type I IFN bioactivity as described.

### Plasmid DNA Immunizations and H5N1 Virus Challenge Infection in Chickens

For the vaccination study, a total of 54 four-week-old specific-pathogen-free leghorn chickens (from IVI) were separated into 8 groups of 6 animals and two control groups of 3 animals. Two suboptimal doses of 25 µg or 2.5 µg of plasmid DNA vaccine were selected for intramuscular immunization, based on information from a recent H5N1 DNA vaccination study in chickens [10]. In this latter study, a prime-boost protocol with 50 µg of HA-expressing plasmid per animal was sufficient to induce full protection against a lethal H5N1 infection, whereas 5 µg of HA DNA resulted in partial protection only. Therefore, in the present study, 25 µg or 2.5 µg of monocistronic plasmid DNA vaccine (either HA DNA or the backbone vector pcDNA6-V5-His B) were mixed with 25 µg or 2.5 µg respectively of chMDA5(1-483) or of parental plasmid (pcDNA6-V5-His B ) as control, and injected to groups of 6 chickens/dose and vaccine formulation. The bicistronic expression plasmids carrying the HA and chMDA5(1-483) gene on the same DNA molecule were applied at a total dose of either 25 µg or 2.5 µg of DNA to two groups of 6 chickens/dose. The plasmids were formulated in 200 µl PBS per animal and were administered by intramuscular injection in the chest muscle. Two vaccinations were applied with an interval of 23 days. 20 days after the second vaccination, *i.e.* 43 days after the first immunization, 1 ml of blood was collected from each animal. Three days later (46 days post-immunization [dpi]), the animals vaccinated with monocistronic expression plasmids or control plasmid DNA were challenged by intratracheal instillation of 10^5^ TCID_50_/250 µl per animal of HPAIV Yamaguchi-7/04 virus diluted in PBS, using a blunt-ended cannula. The infected animals were examined daily for clinical signs for a period of 9 days after challenge. The clinical signs were monitored with a score system described previously [58], with minor modifications. A score of 2.5 was attributed to animals that had to be euthanized in a moribund state. Tracheal and cloacal swabs were collected on a daily basis. All animal experiments with H5N1 viruses were conducted under BSL3+ conditions with respirators and full-body protection.

### Enzyme-linked Immunosorbent Assays (ELISA)

H5-specific antibodies in chicken sera were detected with the ID Screen Influenza H5 Competition ELISA kit (IDvet). In order to quantify H5-specific IgY titers by ELISA [59], 96-well immunoplates (Maxisorb, Nunc) were coated with purified baculovirus-derived recombinant influenza H5 protein (100 ng/100 µl per well, a kind gift from Dr. Matthias Müller, IVI, Switzerland) in 50 mM bi-carbonate buffer pH 9.6 overnight at 4°C. The plates were then blocked with 200 µl/well PBS containing 0.2 M NaCl and 10% defatted dry milk at 37°C for 1 h. The wells were washed three times with 300 µl of wash buffer (PBS containing 0.01% Tween-20). Subsequently, 100 µl of serially diluted chicken sera (PBS containing 0.1% Tween-20 and 3.3% defatted dry milk) were added to the wells and the plates were incubated for 2 h at 37°C. After three wash steps, 50 µl/well of horseradish-peroxidase (HRP)-labeled anti-chicken IgY (Promega) diluted 1∶3000 in PBS/0.1% Tween-20/3.3% defatted dry milk were added and the plates were incubated for 1 h at room temperature. After a final washing step, the plates were incubated at room temperature in presence of ABTS substrate containing 0.01% H_2_O_2_. Optical density (OD) values were recorded after 15 minutes at 405 nm. OD values of 2.1 times the background level (mean OD value of sera from naive animals) or higher were defined as positive. The titers were expressed as reciprocal of the end point dilution factor. H5-specific IgA responses were quantified by ELISA using 1∶10 diluted sera and HRP-labeled anti-chicken IgA (Bethyl) diluted 1∶10′000 in PBS/0.1% Tween-20/3.3% defatted dry milk. OD values were recorded after 60 minutes at 405 nm.

### Haemagglutination Inhibition Assay and Virus Neutralization Assay

The HI assay was performed according to OIE standards (http://www.oie.int/fileadmin/Home/eng/Health_standards/tahm/2.03.04_AI.pdf), with minor modifications. Briefly, 25 µl of AIV (8 haemagglutinating units [HAU] of H5N1 Vac-1/04/) was mixed with 25 µl of two-fold serial dilutions of chicken serum in PBS and incubated in plastic V-bottomed 96-well microtitre plates (Maxisorb, Nunc) at room temperature for 30 min. Chicken red blood cells were washed 4 times with PBS, and 50 µl of 1% (v/v) washed red blood cells in PBS were added to each well. After 40 min incubation at room temperature, the HA titer of the serum was determined as the highest serum dilution causing complete inhibition of 8 HAU of virus. The procedure for the influenza virus neutralization assay was performed according to WHO standards (WHO animal influenza manual; http://www.who.int/csr/resources/publications/influenza/whocdscsrncs20025.pdf), using the H5N1 Vac-1/04 virus.

### Viral RNA Isolation and Quantification

RNA was extracted from cloacal and tracheal swabs using the NucleoSpin RNA II extraction kit (Macherey-Nagel) and amplified by real-time RT-PCR using primers and probe as described elsewhere [54], [60]. Quantitative RT-PCR was carried out with a QuantiTect Probe RT-PCR kit (Qiagen) using a 7900HT Fast Real-Time PCR system (Life Technologies). The Ct values were determined from triplicates. For absolute RNA quantification, an internal standard was used based on M1 gene copies.
